# A case report of successful treatment of pyoderma gangrenosum in a patient with autoimmune hepatitis, and review of the literature

**DOI:** 10.1186/s12876-015-0376-1

**Published:** 2015-10-26

**Authors:** Theodoros Androutsakos, Paraskevas Stamopoulos, Kiriaki Aroni, Gregorios Hatzis

**Affiliations:** Pathophysiology Department, Laiko General Hospital, National and Kapodistrian University of Athens, Medical School, Athens, Greece; Second Department of Propedeutic Surgery, Laiko General Hospital, National and Kapodistrian University of Athens, Medical School, Athens, Greece; Department of Dermatopathology, Medical School, National and Kapodistrian University of Athens, Athens, Greece

**Keywords:** Autoimmune hepatitis, Pyoderma gangrenosum, Methotrexate, Cyclosporine

## Abstract

**Background:**

Pyoderma Gangrenosum (PG) is a cutaneous condition, its diagnosis suggested by the presence of a painful cutaneous ulcer showing rapid progression. Pyoderma gangrenosum is associated with a concomitant systemic disease in 50 to 70 % of cases, including inflammatory bowel disease (IBD), rheumatoid arthritis, and lymphoproliferative disorders. Although PG has also been reported with viral hepatitis, it is rarely associated with autoimmune hepatitis.

**Case presentation:**

A 19-year-old Caucasian female, with a prior diagnosis of autoimmune hepatitis (AIH) in remission, presented with bilateral lower limb ulcers 4 years after the diagnosis of AIH. She was diagnosed with PG and treated with high-dose prednisolone, methotrexate and cyclosporine. One year later she was well, the ulcers completely healed, and with the autoimmune hepatitis still in remission.

**Conclusion:**

We report a case of autoimmune hepatitis and the subsequent, rarely occurring, extra-hepatic onset of pyoderma gangrenosum, with the AIH in remission, strengthening the association between the two conditions. Since both the AIH and the PG can present serious diagnostic challenges, thus delaying vital therapy, it is important that the development of either prompts us to consider the possibility of the other developing in the future or if already present facilitate its diagnosis, such considerations making the case for a systematic follow up.

## Background

Pyoderma Gangrenosum (PG), classified as a neutrophilic dermatosis, is an ulcerative cutaneous condition, first described in 1930 by Brusting and colleagues [1, 2]. Commonly, the lesions present as tender pustules that evolve into enlarging suppurative ulcers, with a tendency to last for months or years.

Although of non-infectious etiology, the pathogenesis of the disease is unclear, but evidence suggests an underlying defective neutrophilic function [1, 3]. After excluding more common causes of cutaneous ulcerations, the diagnosis of PG is suggested by the presence of a painful, necrolytic cutaneous ulcer, with an irregular undermined border, showing rapid progression [1]. Histologically, there is lymphocytic infiltration in the early stages followed by neutrophilic infiltration and hemorrhage [4, 5]. Pyoderma gangrenosum is associated with a concomitant disease in 50 to 70 % of cases, including inflammatory bowel disease (IBD) in 10 to 15 % [6], rheumatoid arthritis [7], and lymphoproliferative disorders [8–10]. Although PG has been encountered with viral hepatitis [11], only a small number of cases have been reported with autoimmune hepatitis (AIH) [12–15]—one associated with concurrent primary sclerosing cholangitis and ulcerative colitis [13].

Treatment of PG is non-surgical and consists of a combination of local wound care and systemic therapy, the latter centered on the use of high-dose corticosteroids [16]. Other treatment agents include immunosuppressants, intravenous immunoglobulin, and biologic agents—such as tumor necrosis factor alpha inhibitors [16]. We report a 19-year-old patient, with a history of AIH in remission, who presented with pyoderma gangrenosum of the lower extremities, and was treated successfully with cortisone, cyclosporine and methotrexate.

## Case presentation

### Clinical aspects

A 19-year-old Caucasian female presented to us with bilateral lower limb ulcers. She reported a scratch on her left ankle 3 months earlier that worsened over time into an ulcer, in spite of antibiotic treatment. A week later, new lesions appeared on the left leg and similar ones were seen on her right leg. The patient denied fever, weight loss, or other signs or symptoms of systemic illness. Four years earlier she had been admitted at another hospital with transaminasemia and jaundice and was diagnosed with autoimmune hepatitis based on serological (ANA and ASMA) positivity and compatible histopathological features, in the absence of viral markers. Treatment with prednisolone and azathioprine brought the disease into remission. The patient was on 5 mg prednisolone upon presentation.

**Fig. 1:**
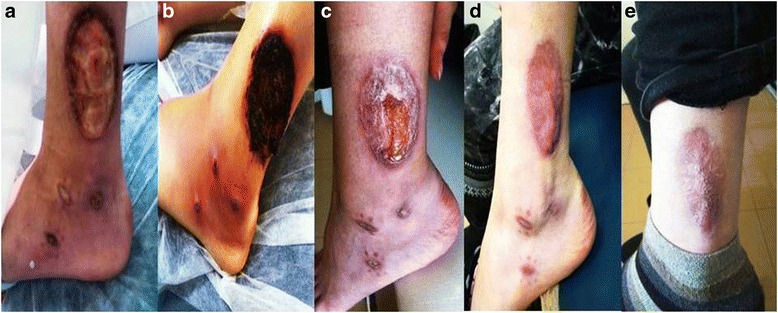
Images of ulcerated areas. **a** On admission. **b** A week later. The swelling and the inflammation are noted. **c** One month later. Significant improvement of the ulcerating areas. **d** At 6 months. The inflammation has subsided, with further improvement of the ulcerating areas. **e** At one year. No new ulcers and healed old ones

On admission, the patient was afebrile, bearing purulent, painful ulcers in both legs (Fig. 1a). Lab results showed the inflammation markers just exceeding the upper normal limits (Table 1). Transaminases were within a normal range, while anti-nuclear antibodies (ANA) were positive at a titer of 1/320 and anti-smooth muscle antibodies (ASMA) were positive at a low titer (1/80). Cryoglobulin test was negative. The lesions were cultured for bacteria and Mycobacterium tuberculosis, and empiric therapy was started with clindamycin and moxifloxacin. Magnetic Resonance Imaging (MRI) of the region of ulcers in both legs revealed diffuse subcutaneous oedema and subcutaneous nodular lesions on the dorsal surface of the feet. With a working diagnosis of pyoderma gangrenosum, a skin biopsy was acquired, her previous medication of azathioprine was reintroduced at 150 mg, while the steroids were increased from 5 to 20 mg prednisolone, daily. The patient was discharged pending the pathology and bacteriology results.

**Table 1:** Patient’s lab results

Lab test	Normal values	Result
Hgb (g/dl)	12,0–16,0	12,4
Hct (%)	38–47	37,7
WBC (K/μL)	4,5–11,0	12,300
Neut/Lymph/Mono/Eos (%)		69/25/5/0
PLT (K/μL)	140–440	357
Glu (mg/dl)	74–106	75
Urea (mg/dl)	15–40	25
Creatinine (mg/dl)	0,6–1,1	0,7
AST (U/L)	5–31	13
ALT (U/L)	5–34	11
γGT (U/L)	7–36	12
ALP (U/L)	48–141	59
INR	0,9–1,15	1
HBA1c (%)	4,8–6	5,5
TSH (mU/L)	0,3–4	3
FT4 (pmol/L)	10–25	17
Total Protein (g/dl)	6,4–8,3	7
Albumin (g/dl)	3,5–5,2	4,1
Total Bilirubin (mg/dl)	0,3–1,2	0,47
Direct Bilirubin (mg/dl)	0–0,3	0,14
Sodium (mMol/L)	136–145	144
Potassium (mMol/L)	3,5–5,1	4,2
CRP (mg/dl)	0–5	15,3
ESR (mm)	0–20	38

**Fig. 2:**
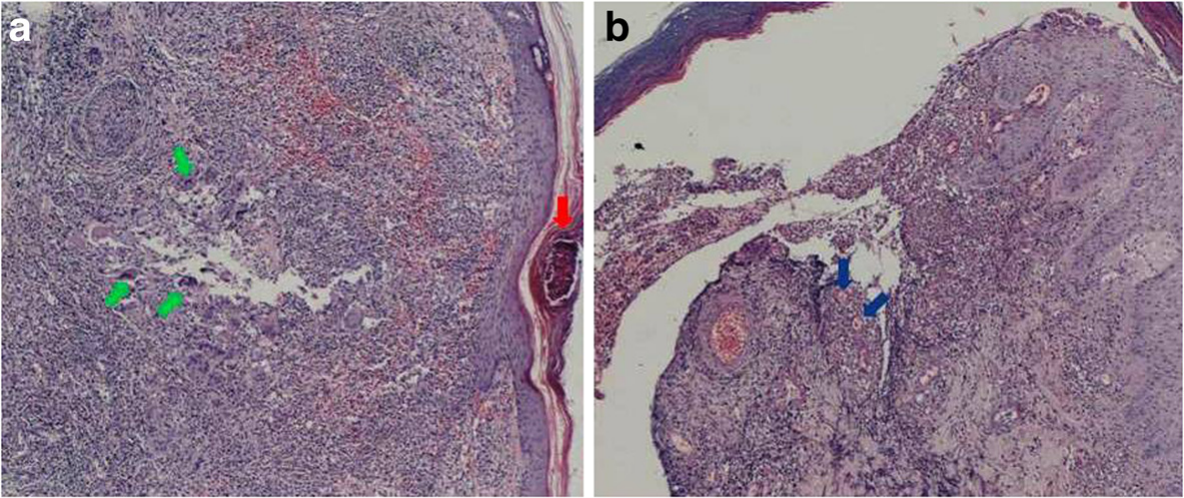
Images of skin biopsy. **a** Abscess formation in the epidermis (red arrow) with accumulation of neutrophils and granuloma (green arrows) formation in the dermis. **b** The edge of an ulcer: deposits of eosinophilic material on the wall of the small blood vessels (blue arrow)

She returned a week later with arthritis of both ankles and worsening of the ulcers (Fig. 1b). Further cultures were again negative. The biopsy was interpreted as being compatible with pyoderma gangrenosum (Fig. 2a, b), hence prednisolone 1 mg/kg was started, azathioprine was switched to cyclosporine at 150 mg that was further increased to 250 mg daily, and methotrexate 10 mg/week was added to the regimen. Given the rare combination of pyoderma gangrenosum with autoimmune hepatitis, upper and lower endoscopies, as well as upper abdominal MRI and Magnetic Resonance Cholangio-pancreatography (MRCP) were done to exclude more common associated diseases. All of the investigations were negative. A review of the initial liver biopsies confirmed the diagnosis of autoimmune hepatitis, showing dense portal and periportal lymphocytic and plasmacytic infiltrates, as well as mild periportal fibrosis. The patient was again discharged, with slow tapering of the steroids.

On follow up a month later she was feeling well, with no ankle swelling, and with all ulcers healing satisfactorily (Fig. 1c). Six months hence she was on 4 mg methylprednisolone, 100 mg cyclosporine, and 7.5 mg methotrexate, with normal liver enzymes, and with the ulcers nearly healed (Fig. 1d). Cyclosporine was discontinued at 8 months, and a year later the patients was on 4 mg methylprednisolone and 7.5 mg methotrexate, with further improvement and complete healing of the ulcers (Fig. 1e).

### Commentary

Autoimmune hepatitis is a chronic self-perpetuating inflammatory disease occurring in all ages and races [17]. Besides its diverse presentation and heterogeneity of the clinical features, AIH is characterized biochemically by elevated transaminases, histologically by interface hepatitis, and serologically by increased levels of immunoglobulin G (IgG) and autoantibodies, in the absence of viral markers [17]. Based on the autoantibody profile, two types of AIH are recognized: type 1 (AIH-1), positive for ANA and/or anti-smooth muscle antibody (SMA), and occurring at any age, and type 2 (AIH-2), positive for anti-liver-kidney microsomal antibody (anti-LKM) or for anti-liver cytosol type-1 antibody (anti-LC-1), with a peak incidence in children and adolescents [17–19]. AIH generally responds to immunosuppressive treatment, but if left untreated usually progresses to liver failure requiring transplantation [17].

Extra-hepatic disorders, mainly autoimmune conditions, are common in autoimmune hepatitis and occur in all stages of liver disease, including ulcerative colitis, Crohn’s disease, vasculitis, arthritis, thyroiditis, diabetes mellitus, autoimmune hemolytic anemia, glomerulonephritis, fibrosing alveolitis, systemic lupus erythematosus, coeliac disease, sicca syndrome, vitiligo, or lymphoproliferative syndromes [20, 21].

On the other hand, PG is associated with a variety of mainly autoimmune diseases in 50–70 % of cases, with inflammatory bowel disease (IBD) topping the list at 10–15 % [6]. However, there is a dearth of data on the association of PG with AIH, as a search of the English-language literature unearthed only fifteen other cases [15].

Our patient had AIH-1, as the majority of reported cases when the type of AIH was identified, although AIH-2 may be underestimated [15]. Our patient’s gender, the age at diagnosis of AIH, and the interval to the subsequent onset of PG concur with those of the other reported cases, as does the development of PG during a quiescent stage of the AIH. Our patient remained in remission subsequently, even with the use of potentially hepatotoxic agents. Although a fulminant presentation of AIH has been proposed as a possible risk factor for the subsequent development of PG [15], our patient’s hepatitis course was not severe, emphasizing our poor understanding of the pathogenesis of PG in relation to AIH. Finally, pathergy, although rare [15], is a hallmark of PG, and our patient’s first appearance of an ulcer was at a previously “scratched” area, raising the distinct possibility of such event.

## Conclusion

In summary, we report a patient with autoimmune hepatitis and pyoderma gangrenosum treated successfully with cortisone, cyclosporine, and methotrexate. Thorough testing excluded concurrent autoimmune diseases, confirming the association of pyoderma gangrenosum with autoimmune hepatitis, even when the latter is inactive. Clearly, the diagnosis of PG should raise the possibility of concomitant AIH, since its diagnosis can be challenging and the results of failure to treat it devastating. On the other hand, AIH should sensitize us to the possible future development of PG. Overall, our case adds to the pool of knowledge about the presentation and management of PG in association with AIH.

### Consent

Written consent was obtained from the patient for publication of this Case report and any accompanying images. A copy of the written consent is available for review by the Editor of this journal.

## Abbreviations

PG: Pyoderma gangrenosum
IBD: Inflammatory bowel disease
AIH: Autoimmune hepatitis
ANA: Anti-nuclear antibodies
ASMA: Anti-smooth muscle antibodies
MRI: Magnetic resonance imaging
MRCP: Magnetic resonance cholangiopancreatography
Anti-LKM: Anti-liver kidney microsomal antibodies
Anti-LC-1: Anti-liver cytosol type 1 antibodies
IgG: Immunoglobulin G
